# Movement synchrony and familiarity in dyadic and four-person conversations

**DOI:** 10.3389/fpsyg.2026.1744354

**Published:** 2026-09-23

**Authors:** Danny Dirker, Anja Stukenbrock, Wolfgang Tschacher

**Affiliations:** 1 Department of German Studies, Heidelberg University, Heidelberg, Germany; 2 University Hospital of Psychiatry and Psychotherapy, University of Bern, Bern, Switzerland

**Keywords:** affect, embodiment, familiarity, social interaction, synchrony

## Abstract

This paper investigates movement synchrony between German-speaking participants in spontaneous verbal interaction. In contrast to previous work where interpersonal synchrony was mostly examined in two-person interactions, we studied both dyads (*n* = 49) and quartets (*n* = 25) of participants, yielding new insights into how bodily coordination emerges in multi-party settings. Drawing on Motion Energy Analysis as a widely recognized video-based method for motion capture, we computed synchrony using windowed cross-correlations, finding that movement synchrony was present in both dyadic and quartet conversations. Furthermore, we found correlational evidence that familiarity between participants contributes to movement synchrony in two-person interactions, as well as to the extent to which individuals synchronize with others in quartets. Additionally, participants’ self-reported affectivity is related to their proclivity to synchronize with their dyadic partner. These findings demonstrate how individual and relationship-level factors shape movement synchrony in both dyadic and quartet informal interactions.

## Introduction

Synchrony–or synchronization–is considered to constitute “a fundamental form of coordination observed across living systems” (Lucherini Angeletti et al., 2025, p. 3; Pikovsky et al., 2001). When humans engage in copresent interaction, they are usually unaware that their nonverbal and somatic responses tend to align spontaneously (see Wiltshire et al., 2020, for a review). This phenomenon has been termed *interpersonal synchrony* (Mayo and Gordon, 2020) and is assumed to function as a “social glue” (Gordon et al., 2025, p. 681), though the question of how exactly it underlies social bonding experiences in human interaction remains open.

While interpersonal synchrony has been widely studied in dyads, synchrony in unstructured multi-party interaction remains largely unexplored. Research on synchrony in groups larger than two has primarily focused on structured activities such as singing, dancing, drumming, marching, etc. (McNeill, 1995; see below). Wiltermuth and Heath (2009) showed that instructed synchrony with others increased cooperation in subsequent group tasks, even when cooperation entailed individual costs. Investigating informal conversations in quartets offers novel insights into how bodily synchrony emerges in multi-party settings and how it relates to social relationships—an endeavor we pursue in the present study by analyzing movement synchrony between both dyads and groups of four participants (‘quartets’) in spontaneous conversations.

From an evolutionary perspective, humans rely on embodied and vocal means to synchronize and coordinate bodies and minds in order to cooperate and collaborate. Beyond goal-oriented activities such as territorial defense, hunting, and managing intergroup conflicts, coordination and synchrony also contribute to social bonding among group members, drawing on mechanisms shared with our closest relatives, the non-human primates. Studies on rhythmic abilities in humans and non-human primates showed that primates can perceive auditory patterns and synchronize motor behavior with external rhythms (see Hattori, 2021, for a review). It has been argued that these mechanisms became particularly important for fostering and maintaining social relationships when hominid group size increased, as the need for practices beyond proximal embodied practices such as touching and grooming arose (Dunbar, 1996). For instance, rhythmic activities such as social laughter, dancing, and singing provide auditory cues that enable “vocal grooming” (Aiello and Dunbar, 1993).

Within this broader framework, Dunbar (2012) discussed two functional explanations for the evolution of music: sexual selection vs. social bonding in groups exceeding the size supported by primate grooming. Musical activities may stimulate the release of endorphins, thus serving as “a form of ‘grooming-at-a-distance’“(p. 209) and strengthening individuals’ group-orientation. He further suggested that laughter may represent an earlier precursor to musical chorusing, as both exhibit segmentation and synchrony. Music may therefore have served as an intermediate stage between laughter and language in the evolution of social bonding mechanisms. Extending theories of music’s evolutionary function in contexts such as mate selection, parental care, coalition signaling, and group cohesion, two recent *BBS* target articles offer contrasting views. Savage et al. (2021) synthesize previous context-specific theories by proposing an overarching “music and social bonding hypothesis” (p. 1). They argue that “social bonding [is] the primary factor shaping the evolution of human musicality” (Savage et al., 2021, p. 16), which enabled bonding at larger group scales than earlier primate mechanisms. By contrast, Mehr et al. (2021) suggest that music may have originated as a form of credible signaling in contexts of infant care and group interaction, communicating attention, commitment, and coalition strength. Despite differing evolutionary trajectories, both accounts emphasize the importance of coordinated behavior in human groups, suggesting that synchrony—whether in vocal, musical, or other social activities—plays a key role in facilitating cohesion, cooperation, and shared orientation among multiple participants. Examining how bodily synchrony emerges in everyday conversations in larger groups thus offers a window into fundamental human capacities for social coordination and bonding.

Most synchrony research in humans has been undertaken in psychology, particularly in developmental psychology and psychotherapy research, where the concept was originally developed (Di Mascio et al., 1957; Scheidt et al., 2021). Since the 2000s, work on interpersonal synchrony has rapidly increased, spurred by research interest across fields such as social (Tschacher et al., 2014; Richardson et al., 2014; Chierchia and Coricelli, 2015), developmental (Leclère et al., 2014; Trainor and Cirelli, 2015; Kirschner and Tomasello, 2010), and clinical psychology/psychiatry (Ramseyer and Tschacher, 2006; Marci and Orr, 2006; Fuchs, 2015), psycholinguistics (Pickering and Garrod, 2004; Kootstra et al., 2020), cognitive science (Çakır and Huckauf, 2024; Louwerse et al., 2012; Oben and Brône, 2016; see Zhao et al., 2024 for a review of fNIRS hyperscanning; Drijvers and Holler, 2022 dual-EEG), empirical aesthetics (Tschacher et al., 2023), phenomenology (De Jaegher and Di Paolo, 2007), and conversation analysis (CA)/interactional linguistics (IL) (e.g., Auer, 2005, 2024; Goodwin, 1980, 2007; Goodwin and Goodwin, 1987; Mondada, 2018; Streeck and Jordan, 2009; Stukenbrock, 2020, 2023).

### A selective overview of key findings in conversation analysis (CA)

To date, with few exceptions (Auer, 2024; Deppermann and Streeck, 2018; Ehmer, 2021; Harjunpää and Voutilainen, 2025; Pfänder and Couper-Kuhlen, 2019; Stukenbrock, 2018a, 2021), the terms *synchronization* and *synchrony* are rarely used in CA. Over the past decades, this line of research has developed growing interest in *Gestalt*-like, multimodal assemblages of verbal and bodily means of communication, where *multimodal* refers to phenomena co-occurring in different modalities such as speech, gaze, gestures, facial expressions, body posture and body movements. Researchers in this domain have favored terms such as *coordination*, *simultaneity*, *quasi-simultaneity*, and *co-temporality* (Stukenbrock, 2015, pp. 28–35). Seminal edited volumes in the field have highlighted that multiple temporalities—operating at different levels of granularity (Deppermann and Günthner, 2015; Deppermann and Streeck, 2018; Streeck et al., 2011)—are involved in the intra- and interpersonal coordination (Deppermann and Schmitt, 2007) of verbal and bodily means of communication.

Various studies have examined participants’ synchrony of rhythmic and prosodic features in spoken interaction. Based on an audio corpus of German telephone conversations, Auer (1990) showed that both interlocutors contributed to the construction of rhythmically isochronous structures to navigate from pre-closing to the termination of phone calls. Isochrony was defined as the equal spacing between prosodically emphasized syllables, and tempo as “the duration of isochronous intervals” (Auer, 1990, p. 362). Subsequent work empirically explored the interplay between rhythm and tempo of conversational structuring in different languages such as English (Couper-Kuhlen, 1993), German and Italian (Auer et al., 1999).

Early studies on the coordinated interplay between verbal and embodied practices reported “movement mirroring” (Kendon, 1970: 113) between speaker and addressee as opposed to co-listeners, as well as micro-adjustments between speakers and recipients during utterance construction (Kendon, 1967; Goodwin, 1981). It was observed that head nods (Birdwhistell, 1970) and other recipient tokens (Kendon, 1967; Goodwin, 1981) are consequential for the emergence of the speaker’s turn. Subsequent studies found that recipients’ head nods during storytelling displayed affiliation with the storyteller (Stivers, 2008; Voutilainen et al., 2019), and that head nods in response to questions served nodders to answer without claiming speakership and thus enabled the questioner to continue (De Stefani, 2021).

In face-to-face interaction, the coordination of speech units, facial expressions, gestures, body postures and movements relies on the participants’ visual access to one another, which facilitates movement synchrony while also being reinforced by synchrony. The mutuality of visual perception is enacted by speakers’ and addressees’ attention to the gaze of their interlocutor. Pioneering studies by Kendon (1967), Argyle and Cook (1976) and Goodwin (1980) established the importance of gaze for turn-taking and the negotiation of participation. It was found that recipients typically look at the speaker, while speakers alternate between gazing at and gazing away from their interlocutors. Early studies of dyadic interaction showed that speakers often avert their gaze at the beginning of a turn and return it toward addressees near its end, a pattern interpreted as turn-regulatory (Kendon, 1967) or as reflecting the cognitive demands of utterance planning (Beattie, 1978, 1979). Goodwin (1980) emphasized the interactional function of gaze showing how speakers actively pursue mutual gaze with their addressees at turn beginnings.

In a different vein, Streeck (1993) focused on the function of speaker gaze toward simultaneous gestures during social interaction. He observed that temporal coupling of “so”/"like this” with speaker gaze directed at the speaker’s own gestures draws addressees’ attention to those gestures. This observation on the interactional synchrony between speaker gaze, gesture, and addressee gaze was refined by Stukenbrock (2015), who showed that this occurs only when the modal particle “so” is produced with prosodic stress (see also Ningelgen and Auer, 2017). In a mobile eye-tracking study on the combined use of “so” with non-lexical, rhythmic vocalizations during music rehearsals, Balantani (2022) demonstrated that co-performers’ rhythmic demonstrations served joint decision making on how next pieces should be played. Extending video-based studies on deixis and joint attention, Stukenbrock (2018a,b) deployed mobile eye-tracking glasses to uncover systematic gaze patterns of speaker and addressee that serve to synchronize mutual perception as well as joint attention to objects in demonstrative reference. Work on speakers’ micro-timing of requests for addressees’ attention in museums documented the strategic deployment of gaze requests to delay or accelerate joint onward movement (Stukenbrock, 2023).

Mobile eye-tracking evidence demonstrates that interactants coordinate the precise timing of gaze shifts (Zima and Stukenbrock, 2025) with ongoing body movements and talk. This supports the view that interpersonal synchrony is not limited to body movement alone but emerges as interactants engage in coordination efforts across multiple organizational levels and modalities—body movement, visual perception, and talk. These efforts unfold through intersecting trajectories of simultaneous (i.e., synchronized), serial and sequential activities and actions.

### Empirical review of movement synchrony

Several issues challenge research on synchrony within and across disciplines, including terminological inconsistencies^1^–such as the interchangeable use of *synchronization* and *coordination*, although *coordination* denotes a broader conceptual domain, whereas *synchrony* refers more narrowly to temporal alignment. Concerning the problem of terminological inconsistency (Wiltshire et al., 2020; Scheidt et al., 2021), we understand *coordination* as the broader concept encompassing various forms of interactive alignment between participants or living beings in social interaction. Within this framework, *synchrony* constitutes a more specific, temporally grounded form of coordination—referring to the precise timing (*chronos*, χρόνος) of behaviors, movements, or signals. Synchrony is thus particularly concerned with temporal dynamics, such as rhythm and simultaneity in interpersonal interaction. The concept is closely related to measurable time and mostly applied to quantifiable phenomena evaluated by external measurements (Tschacher et al., 2018). Nowadays, technological advances enable unobtrusive, real-time recordings of spontaneous interaction, allowing for the acquisition of high-precision data on various types of synchrony with minimal compromise to ecological validity.^2^ The present study examined interpersonal coordination on the behavioral level by analyzing movement synchrony of the whole body. We consider movement synchrony to occur when two or more interactants move their bodies simultaneously or with a short time lag, for instance, when changing postures, gesturing, moving their heads and/or limbs, etc.

In a recent theoretical paper, Gordon et al. (2025) postulated that interpersonal synchrony arises because we are pulled “to synchronize and segregate” (p. 685) from one another. Based on extensive review of experimental and observational evidence, they propose that participants yield to these pulls to synchronize (or not) with their current social partner(s) due to central characteristics related to the individual (e.g., gender, affect, age, personality traits), the relationship (e.g., gender configuration, mutual familiarity), and/or context (setting: competitive vs. cooperative). Importantly, these variables may interact in how they influence movement synchrony (see also Chartrand and Lakin, 2013). For example, while opposite-gender dyads may exhibit more distinct synchrony compared to same-gender dyads, this may change when participants are younger vs. older, or friends vs. strangers. Experimental findings indicate that synchrony was sometimes found to be stronger in competitive as opposed to cooperative interactions (Tschacher et al., 2014) as well as in therapeutic relationships leading to drop-out (Paulick et al., 2018). These findings underscore that movement synchrony between participants is shaped by multiple interacting individual, relational, and contextual factors. Against this backdrop, the present study examines individual-level variables (age, current affect) as well as relationship-level variables (familiarity) and how they relate to participants’ movement synchrony in spontaneous talk.

Among these factors, familiarity and affect have been considered by various research groups. Tschacher et al. (2014) found that in same-gender stranger dyads (*n* = 84), affect ratings were positively associated with movement synchrony, such that the stronger the observed synchrony, the more positive was the individual post-interaction affect. Interestingly, individual pre-interaction affect did not predict the degree of synchrony despite modulatory effects on affect ratings of personality traits (e.g., being ‘introverted-distressed’) and interaction type (e.g., competition, cooperation). Likewise, Gordon et al. (2025) conducted an empirical study of movement synchrony in dyads who performed choreographies of varying congruency (critical condition) with a partner via a computer-mediated live video call. Among several psychological traits examined, empathy, emotional contagion and agreeableness significantly interacted with condition to predict higher levels of movement synchrony, leading to more similar task performance across conditions. Specifically, individuals with higher scores on these personality traits synchronized their body movements with their dyad partners’ more effectively. A potential mechanism linking affect to movement synchrony is that positive affect enhances prosocial attention toward others (Aknin et al., 2018; Snippe et al., 2018). Accordingly, experiencing higher levels of positive affect may foster a prosocial orientation in which participants—whether in dyads or quartets—more readily exhibit synchrony, underscoring our hypothesis that interpersonal synchrony is positively associated with affectivity (H3).

Beyond affectivity, relationship quality—particularly the degree of familiarity between interactants—represents another factor that may shape the emergence of movement synchrony. Relationship quality typically reflects mutual familiarity, trust, empathy, and shared goals—factors that facilitate synchronization between participants. In clinical counseling and therapeutic settings, Ramseyer and Tschacher (2011) found that improvements in relationship quality over the course of treatment were associated with increases in movement synchrony. Wiltshire et al. (2020) observed that in dyadic psychotherapy, interpersonal coordination between therapists and clients in the domains of bodily movements, interpersonal processes, physiology and vocalizations or speech patterns were related to psychotherapy outcomes, therapeutic alliance and reports on empathy across different therapeutic approaches.

Although psychotherapy provides a rich context for studying the link between relationship quality and synchrony, similar mechanisms are likely to operate in everyday interaction, where relationship quality is reflected in the degree of mutual acquaintance, that is, familiarity, between participants. To operationalize this concept in our conversational setting, participants rated how well they knew one another on a 5-point Likert scale, with familiarity serving as a graded proxy for relationship quality. As such, familiarity captures key dimensions of relationship quality, including prior shared experience, trust, empathy, and alignment of goals. For such non-clinical settings, research on familiarity and movement synchrony is scarce (cf. Table 4 in Rennung and Göritz, 2016). Feyereisen (1994) reviewed qualitative research documenting differences in body movement between friends and strangers. Latif et al. (2014) investigated friend-or-stranger casual dyadic conversations and found that the friend-sample exhibited significantly higher levels of movement synchrony compared to the stranger sample, although synchrony was present in the stranger-sample as well (cf. Tschacher et al., 2014). More recently, Gordon et al. (2025) reviewed empirical evidence on synchrony across multiple modalities and concluded that the effects of familiarity may vary across interactional contexts. For instance, eye contact among friends was linked to higher levels of brain synchrony as compared to strangers (Luft et al., 2022).

Building on evidence from psychotherapy (Wiltshire et al., 2020; Ramseyer and Tschacher, 2011), and contributing to the ongoing debate on the role of familiarity in interpersonal synchrony, we treat pre-existing familiarity as a proxy for relationship quality and predict that higher ratings of familiarity will be associated with higher levels of movement synchrony (H2).

### Movement synchrony in dyads and in groups

Much of the reviewed research has observed synchrony in dyads (i.e., two-person interactions, the smallest social aggregate) and in controlled laboratory settings. In line with previous work on psychotherapy, synchrony is considered to fulfill important functions with respect to joint attention, interactional alignment and affiliation, cooperation and collaboration. The present study contributes to the existing body of research more broadly—beyond the laboratory and the institutionally specific realm of psychotherapy research—to understanding movement synchrony as a dimension of interpersonal coordination in two distinct participation frameworks, dyads and quartets. Whereas dyads have been extensively studied before, to our knowledge, this is the first analysis of movement synchrony in informal interactions between quartets.

From an interactional perspective, quartets are particularly interesting for two reasons. First, they constitute participation frameworks which lend themselves to forms of verbal and embodied activities termed “byplay,” defined as “subordinate communication of a subset of ratified participants” (Goffman, 1981, p. 134). Their participation framework differs from “crossplay […] between ratified participants and bystanders across the boundaries of the dominant encounter,” and “sideplay” exclusively arising among bystanders (Goffman, 1981, p. 134). Byplay occurs simultaneously to the current flow of talk and may take the form of “asides, parenthetical remarks, and even quips” (Goffman, 1981, p. 29; cf. Goodwin, 1990, on collusive forms of byplay). While this kind of subordinate communication also occurs in triadic interaction, triadic participation frameworks do not lend themselves to interactional schisms. By contrast, conversation among more than three participants, i.e., multi-party interaction, may occasionally split off (Goffman, 1963) into two or more different threads which are sustained by sub-groups of participants. Moving from dyads to quartets thus entails the possibility to study the local variability of participation frameworks, how they are achieved, and the role of embodied conduct in the emergence and dissolution of varying participant configurations (Kendon, 1970).

From an analytical perspective, multi-party configurations allow for intra-group comparisons of variables contributing to movement synchrony. For instance, dyadic synchrony measured in two-person interactions may be compared to the synchrony of corresponding dyads nested in quartets, which enables estimations of dyad-specific variance. Importantly, nested dyad-level measures bear potential effects due to the degree of mutual familiarity, gender configuration, or shared interactional history. For example, two familiar peers in a quartet may exhibit stronger movement synchrony within their nested dyad than with the other, less familiar participants. In addition, the potentially variable impact of individual-level factors conducive to synchrony (such as age, gender, or current affect) can be examined across participant frameworks. As such, quartets permit tests of putative social functions of synchrony—such as rapport, bonding, or the prioritization of particular interlocutors (cf. focus persons; Deppermann and Schmitt, 2007)—and enable inference about which factors drive observed degrees of movement synchrony, rather than merely documenting that synchrony occurs.

Critically, however, participant configurations beyond the dyad bear limitations upon findings gained from quantitative analyses. For instance, synchrony in quartets can occur in multiple relational configurations: an individual may be synchronized with one, two, all three, or none of the co-participants. This creates far more possible combinations than in dyads, where only one partner is available. As a result, several dyadic or triadic synchrony relations can exist at once, and an individual’s movement may align differently with each co-participant. While this may be beneficial for isolating individual- or dyad-level contributions to synchrony, these varied patterns can cancel out effects at the group level because the computation treats all members’ data streams equally and yields a single synchrony measure. The quartet therefore constitutes a new and more complex interactional system that develops its own dynamics. In quartet conversations, there is a greater chance of talk-in-overlap, difficulties in floor management, shifting participation status, spatial orientational changes, to name a few. In terms of cognitive demand, there are more speakers to comprehend, likely more assertions made, more positions to remember, more co-participants to convince, etc. Interaction becomes more complex when three instead of one co-participant need to be taken into account. In sum, the examination of four person conversations introduces participation structures, relational configurations, and cognitive demands that are absent in dyads, thereby enabling the investigation of interactional dynamics that do not emerge in two-person contexts.

To date, only few studies have examined nonverbal interpersonal synchrony between more than two participants. Meier and Tschacher (2021) computed multivariate synchrony on simulated and empirical data, finding that movement synchrony of groups was reliably detected using multivariate surrogate synchrony computation. In a laboratory study, Gordon et al. (2020) instructed triads to drum in sync and detected cross-modal synchrony of body movements with inter-heartbeat intervals between triad members. Frank and Richardson (2010) measured rocking-chair movement synchrony in small groups (5 participants each) and demonstrated the feasibility of a Kuramoto order parameter of phase synchronization as a robust statistic for synchrony measurement in multi-party configurations. Tunçgenç and Cohen (2016) split groups of six 7–10 year olds into in-group vs. out-group-triads and instructed them to perform a simple, pre-studied choreography to a drum beat. Children’s behavioral and self-reported indices of social bonding with out-group members were significantly increased when the out-group had performed the choreography at the same tempo and rhythmically aligned with the in-group than when they had not, underscoring positive effects of movement synchrony on prosocial attitudes toward strangers. In non-laboratory settings, the predominant interest in developmental psychology is on the parent–child-dyad (see, however, Gordon and Feldman, 2008 on synchrony and co-parenting in the triad), and in psychotherapy on the therapist-client dyad. Recently, Tschacher et al. (2023) investigated large groups of individuals in concert audiences and found that audience members’ subjective aesthetic experiences and emotions were associated with *induction synchrony*^3^ in the context of classical music, based on measurements of body movement and multiple physiological responses (e.g., heart rate, skin conductance, respiration rate).

Without a doubt, these empirical accounts exemplify various research endeavors to study movement synchrony in group settings. Importantly, however, in most of the studies, participants synchronized with each other via an external source, be it through explicit task instruction (Frank and Richardson, 2010; Wiltermuth and Heath, 2009) or music/rhythm (Tschacher et al., 2023; Gordon et al., 2020; Tunçgenç and Cohen, 2016; see also Timmers et al., 2014; Ellamil et al., 2016). Although measured with the same methods, this form of induction synchrony is inherently different from the movement synchrony that arises spontaneously in unstructured social interaction, where it is not induced and participants do not attend to being synchronized. We are unaware of empirical studies that examined movement synchrony in spontaneous interactions of groups larger than two where there was no group-external trigger of synchrony (see, however, Gordon and Feldman, 2008).

Against the background of seminal research on movement synchrony, the present study examines how movement synchrony emerges spontaneously in informal conversation among dyads and quartets, and how it relates to individual affect and participants’ familiarity with one another. Compared with dyads, quartets involve more complex participation structures and relational configurations, giving rise to increased interactional demands. To investigate these dynamics, we engaged groups of four adults in informal conversations while seated together at a round table. This four-person setting established a well-defined condition that ranged between the minimal dyadic and a multi-person group condition.

### Hypotheses

As has been shown, the driving forces of interpersonal synchrony are highly complex, and it is far from clear which factors are the main drivers, to what extent, and with which effect (Wiltshire et al., 2020). This study investigated a small subset of previously identified predictors of movement synchrony by focusing on familiarity and affect and extending previous work on dyadic interactions to include the investigation of bodily synchrony in social interaction among quartets. We asked the following question: Are there differences in movement synchrony in dyads as compared to quartets, considering, first, how participants felt before and after the conversation, and second, how well they knew their co-participant(s)?

*H1*. There is significant interpersonal synchrony of participants’ body movements both in dyads and in quartets.

*H2*. Interpersonal synchrony is positively associated with familiarity between participants in dyads and quartets.

*H3*. Interpersonal synchrony is positively associated with affectivity.

## Methods

### Sample

A total of 100 participants were recruited for the study. Most participants (77 female, 22 male, 1 diverse) were students at Heidelberg University (age: 18–58 years, *M* = 24, SD = 7). All were fluent speakers of German. Monetary compensation was not provided. We targeted lectures and classes at the Faculty of Modern Languages because we were expecting students to be intrinsically motivated, thus recruiting a convenience sample. As we aimed to study conversation in groups, we invited groups of participants rather than individuals. It was emphasized that participation in the study would be casual, and volunteers could choose their peers. Thus, a large percentage of acquainted participants was part of the sample.

### Setup and procedure

Twenty-five groups met the experimenters (male post-graduate researcher D. D., head of Research Unit A. S., and/or one female student assistant) in the mobile eye-tracking laboratory of A. S. at the Department of German Studies of Heidelberg University. Participants were given codes as identifiers, seated at a round table with approximately 1.20 meters distances between the chairs. The experimenters welcomed the participants and obtained written informed consent to undertake audio, video, and mobile eye-tracking recordings. Recordings were then initiated.

Participants completed different tasks throughout the recording (see Figure 1). These tasks included conversations as dyads (interaction 1), as a quartet (interaction 2), and three iterations of an affectivity questionnaire (PANAS). At the beginning, all four participants filled out the first of the PANAS surveys, on which they wrote their code and age. Then, the group was split up into two separate dyads. One of the dyads was taken to a different room, the other stayed. Both dyads were instructed to plan a 5-course-meal made up of courses neither of them liked (a ‘fun task’, see Tschacher et al., 2014). It was clarified that the task was not obligatory but rather an icebreaker and that participants were free to talk about a topic of their choice.^4^ The experimenters left the room. On average, these dyadic interactions (*n* = 49) were ended after 13 min 52 s (SD = 41 s) by the experimenters. The dyads then completed the second PANAS questionnaire and rated how well they knew their dyad partner (on a 5-point Likert scale, familiarity measure) before being reunited as a quartet. As quartets (*n* = 25), they were then tasked with planning a 5-course-menu that everybody liked.^5^ Participants spent on average 14 min 7 s (SD = 1 min 9 s) interacting as quartets. After the quartet interaction ended, participants completed the third PANAS survey. They then rated their familiarity with the two other participants from the quartet with whom they had not interacted in their initial dyads. Upon completion of the third questionnaire, recordings were terminated and participants left.

**Figure 1:**
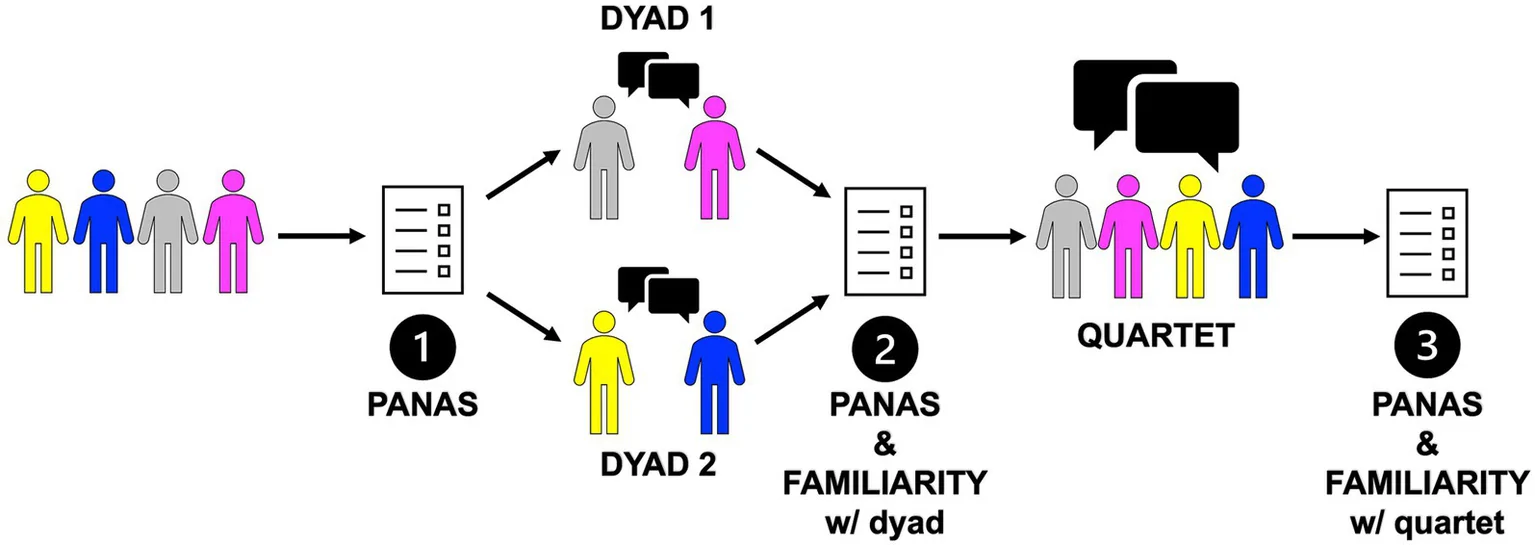
Sketch of the data collection procedure. All participants filled out the first of three PANAS surveys. Then they were split up into dyads and spoke with each other in different rooms. They filled out the second PANAS questionnaire and rated the familiarity with their conversation partner. Then they were reunited and had a conversation as a quartet. At the end, they filled out the third PANAS survey and the members of dyad 1 rated their familiarity with the members of dyad 2 and vice versa.

### Measures of affective states (PANAS)

Participants assessed their current emotional state using a German version of the Positive and Negative Affect Scale (PANAS; Breyer and Bluemke, 2016; Watson et al., 1988). This standardized self-report measure lists 20 adjectives (e.g., ‘active,’ ‘interested,’ ‘upset,’ ‘afraid’), and participants rate on five-point scales (from ‘very slightly’ or ‘not at all’ to ‘extremely’) how much they feel like the adjective says at the moment. The items cluster equally into two affect dimensions: positive and negative affect. The PANAS questionnaire has been extensively validated and is widely regarded as a reliable instrument for assessing affective states (Crawford and Henry, 2004).

### Familiarity ratings

After the interactions, participants assessed how well they knew the other group members on a 5-point Likert scale. This rating was integrated as an additional question on the sheets of PANAS 2 and 3. At the end of PANAS 2 after the dyadic conversation, the question was ‘Do you know the other person?’, with responses ranging from ‘not at all’ (1) to ‘very well’ (5). After the quartet interaction, on PANAS 3, they were asked ‘Do you know the other two people?’ and rated their familiarity with the ‘person across on your left’ and the ‘person across on your right’ separately. The experimenters clarified orally that this referred to the two participants of the opposite dyad, avoiding a further rating of their original dyadic partner. These ratings were treated as measures of familiarity and used as interval-scaled predictors in analyses of movement synchrony.

### Video recording

Data was collected over the course of 1.5 years. Digital video cameras recorded participants’ movement from an external, full-shot perspective in both rooms. In room A, an action camera (*GoPro Hero4*) was attached to a ceiling lamp. In room B, a video camera (*Sony HXR-NX70E*) was placed on a tripod at about 2 meters height and approximately 1.50 meters in front of the participants. The cameras in both rooms offered a full view of the participants’ bodies and their spatial configuration (see Figure 2). These recordings allowed extracting motion energy from video, which requires that moving entities are filmed against a static background (see section ‘Motion Energy Analysis’ below). The present study is based on this video material.

**Figure 2:**
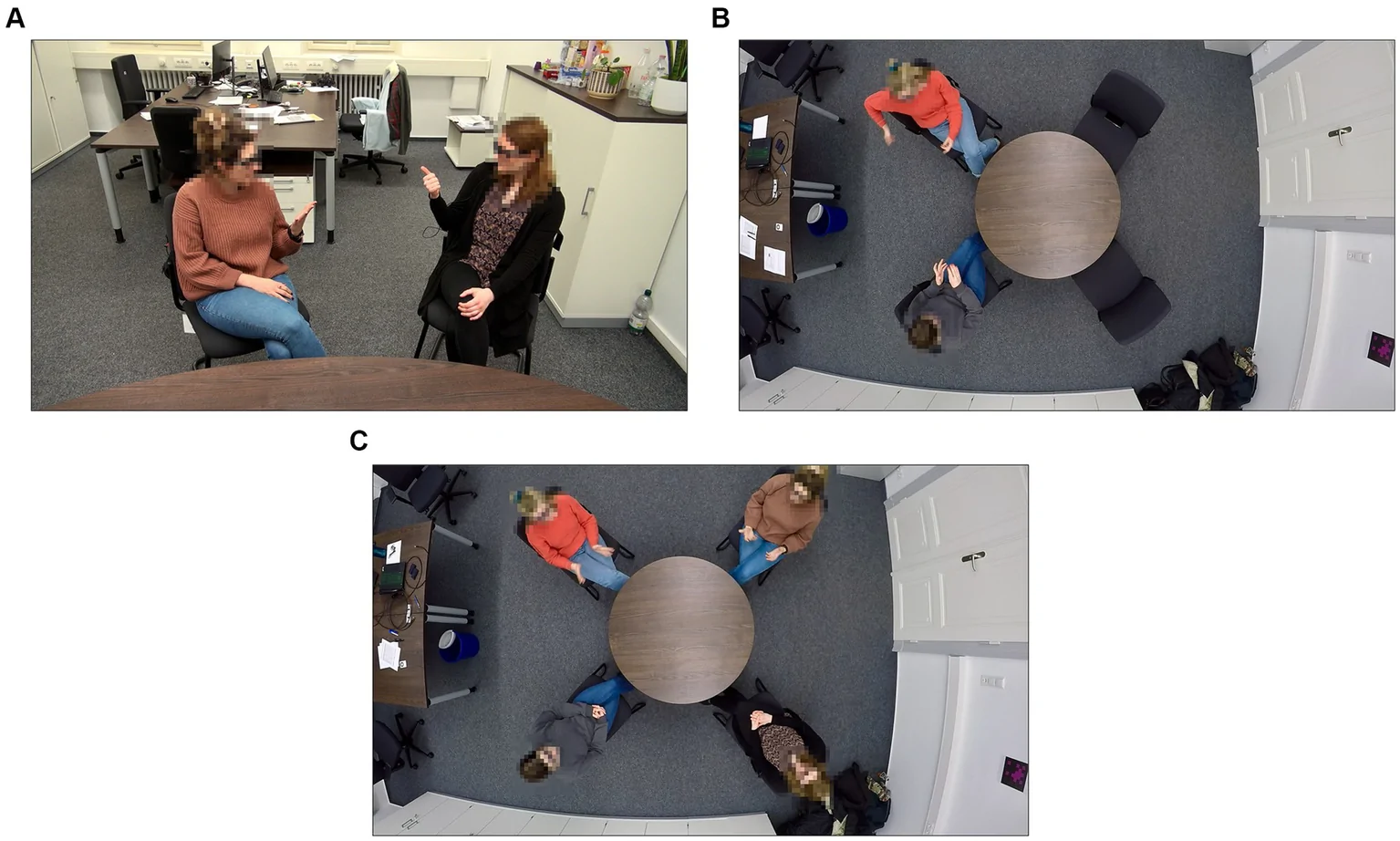
Screenshots of the video recordings. The top row exemplifies the dyadic interactions in two different rooms, with dyad 1 on the left **(A)** and dyad 2 on the right **(B)**. The bottom image **(C)** shows the quartet configuration.

In addition to the collection of movement data, the study also aimed to analyze eye movements and social gaze. To this end, participants wore mobile eye-tracking glasses (Tobii Pro Glasses 2 and 3). Drawing on these gaze data and the accompanying audio recordings, subsequent interaction-analytic studies are planned (cf. Stukenbrock and Zima, 2025; Stukenbrock, 2020) to investigate the interface between movement synchrony, talk, and the multimodal details of social interaction across different participation frameworks. The analyses of verbal interaction and gaze, however, lie beyond the scope of the present study.

### Motion energy analysis (MEA): body movement data

MEA is a frame-differencing algorithm to detect pixel changes in video recordings (Ramseyer and Tschacher, 2011; Grammer et al., 1999). After the respective conversations, the recorded high-resolution raw video files were extracted from the external cameras and imported into MEA (version 4.11b; Ramseyer, 2020). Movement of each participant’s entire body during the interactions was captured. Regions of interest (ROIs) were placed as equally sized areas covering the bodies of the participants and a peripheral area around them to capture all limb motions (see Figure 3). To prevent ROI overlap, participants were seated with approximately 1.20 m distance to each other. The 50 fps (frames per second) video streams resulted in 50 Hz time series of movement energy data, allowing the examination of even minimal motor behavior in high temporal resolution (one sample every 20 milliseconds). Each MEA sample consists of the number of pixel changes encountered since the previous frame.

**Figure 3:**
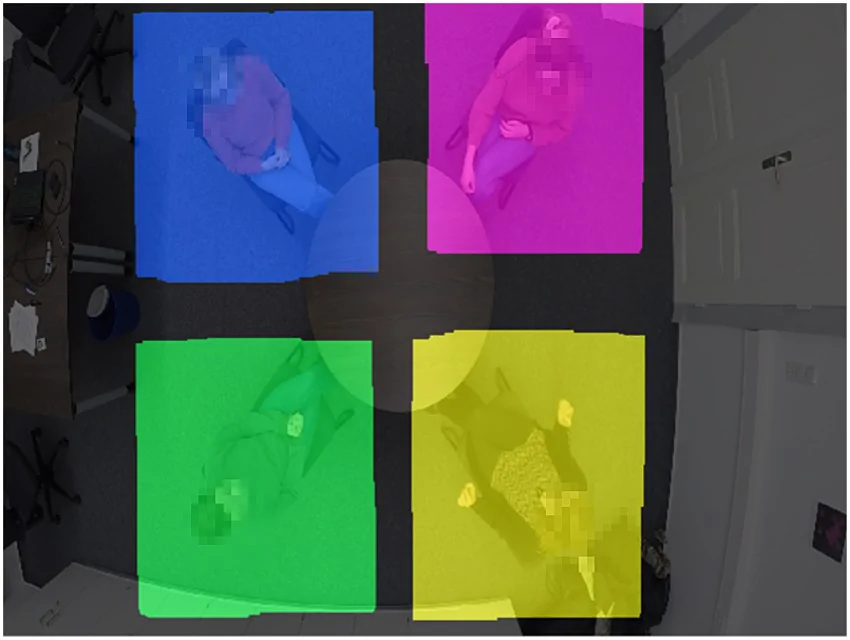
Screenshot from the MEA v4.11b software interface showing regions of interest (ROIs) drawn over each participant’s body in a quartet configuration. Motion energy for each participant is computed from pixel changes within their respective ROI.

### Quantifying movement synchrony: SUSY, mv-SUSY and synchrony contribution

The time-series output of MEA was then processed in RStudio (Posit Team, 2025), using packages *SUSY* (v0.1.1; Tschacher, 2023) and *mvSUSY* (v0.1.0; Tschacher and Meier, 2023) to compute dyadic and quartet measures of movement synchrony.

The SUSY algorithm addresses the cross-correlation of dyadic time series of participant-specific movement energy. The time series are cut into temporal segments. Within each of the non-overlapping segments, the cross-correlations between the time series are calculated up to a certain lag. In the present study, the two parameters segment-size and maximum lag (maxlag) were set to the package’s default values (segment-size: 30 s; maxlag = ±3 s). Cross-correlations are then Fisher’s Z transformed and aggregated as means per segment to yield a measure of per-segment movement synchrony. These means are again aggregated across all segments of the dyad’s time series. This mean-Z of all segments is positive when both participants’ movement data are predominantly positively correlated, suggesting in-phase synchrony overall. It is negative when participants’ movements are negatively correlated, meaning that high movement in one person and low movement in the other co-occurred (anti-phase synchrony). In a second, surrogate step, the sequence of segments of each participant is randomly shuffled and cross-correlation is repeated on such surrogate time series, thus computing cross-correlations of movement that did not happen at the same time. Shuffling is repeated many times^6^ so that a control condition is created, which allows to compute an effect size (ES). The overall mean-Z and standard deviation (SD) obtained from surrogate segments are compared to the mean-Z obtained from the original, aligned data. The effect size is computed as:

$$ES_{\text{noAbs}}=\frac{\left(\bar{Z}-{\bar{Z}}_{\text{SURROGATE}}\right)}{\sigma ({\bar{Z}}_{\text{SURROGATE}})}$$

In some previous studies (e.g., Ramseyer and Tschacher, 2011; Tschacher et al., 2014; Paulick et al., 2018) only absolute values of cross-correlations were considered. In recent years, the default has become to use non-absolute values (*ES_noAbs_*)—as in the present paper—in order to enable distinguishing in-phase from anti-phase synchrony. Further surrogate methods other than segment-shuffling may also be considered such as person-shuffling, which is possible using the *SUSY* package, or methods based on nonlinear dynamics (Schreiber and Schmitz, 2000).

Multivariate surrogate synchrony (mv-SUSY) was applied to the groups with four participants (quartets), where four movement time series were recorded. As a variant of SUSY for multi-person interactions, this method also results in a single measure of synchrony (*ES*) that reflects how aligned all four group members were in their movements. Specifically, mv-SUSY identifies how strongly the movement patterns are coordinated by analyzing the 4 × 4 correlation matrix, with the highest eigenvalue of this matrix (*λmax*) serving as an indicator of synchrony strength. As in SUSY, surrogate analysis is employed in mv-SUSY as a control procedure. The dataset is divided into non-overlapping segments (here: 10 s), and *λmax* is computed for each segment. The group’s empirical synchrony is the average of these segment-wise *λmax* values. To create surrogate datasets, participant time series segments are randomly shuffled, and the eigendecomposition procedure is repeated in the resulting correlation matrices. The number of surrogate datasets drawn and analyzed this way was set to the default of 1,000. The surrogate synchrony (*λmax-surr*) is the mean of these 1,000 surrogate *λmax* values. Ultimately, the effect size (*ES*) is calculated as:

$$\mathrm{ES}=\frac{\left(\bar{\text{λmax}}-{\bar{\text{λmax}}}_{\text{SURROGATE}}\right)}{\sigma ({\bar{\text{λmax}}}_{\text{SURROGATE}})}$$

Considering that the result of both algorithms is a single measure that represents the aggregate movement synchrony of either (nested) dyads or multi-person groups, neither *ES_noAbs_* nor *ES* can be directly analyzed with variables of individual participants, such as self-reported affect or age. To circumvent this issue, individual *synchrony contribution* was computed by applying SUSY to all dyadic permutations nested in a larger group (Tschacher et al., 2023). In quartets, for instance, each participant exhibits movement synchrony with three others, and each permutation forms a unique dyad. Analyzing each of these dyads with SUSY yields multiple *ES_noAbs_*. The average of these values is considered the person’s individual synchrony contribution (*contribES*), representing an estimate of how much that person contributed to the overall group synchrony (see Gordon et al., 2021, for an equivalent procedure in triads).

The described measures were used as dependent variables in the statistical analyses described below. While *ES_noAbs_* reflects the strength of observed movement synchrony of a (nested) dyad, *ES* extends this measure to represent the collective synchrony of the whole multi-person quartet interaction, and *synchrony contribution (contribES)* represents a more fine-grained variable that captures the extent to which the body movement of a single person influenced the synchrony measured in the whole group. Note that due to the specific computational procedure, only *ES_noAbs_* allows for an assessment of synchrony to be in-phasic or anti-phasic.

### Statistical analysis

We used RStudio (Posit Team, 2025) for data processing and JMP Pro 15.1 (SAS Institute Inc.) for statistical analysis. In all statistical models, movement synchrony was the dependent variable (*ES_noAbs_*, *ES*, and *synchrony contribution*). As independent variables we specified familiarity, affect ratings (positive, negative affect), PANAS iteration (before or after the dyad or quartet), and age. For each dataset, we also performed one-sample t-tests against the null hypothesis that the respective synchronies were not different from zero (Hypothesis 1). Mixed-effects regression models were computed to evaluate Hypotheses 2 and 3. Data from 49 dyads and 25 quartets was analyzed.

## Results

*Hypothesis 1*: Presence of movement synchrony in dyadic (SUSY) and quartet (mv-SUSY) interactions.

Movement synchrony in dyads (*n* = 49) is represented by the effect sizes of empirical synchrony against surrogate synchrony from the SUSY algorithm (variable: *ES_noAbs_*). Testing for the above-chance occurrence of synchrony in dyads, a one-sample *t*-test pointed to significant nonverbal synchrony in dyads (*t*(48) = 5.29, *p* < 0.0001). The mean effect size of *ES_noAbs_* was 13.74 (SD = 18.17). This constitutes strong evidence for the presence of interpersonal movement synchrony in the dyad sample. This interpersonal synchrony was in-phasic.

For quartets (*n* = 25), multivariate surrogate synchrony (mv-SUSY) was computed, and effect sizes (*ES* based on *λmax*) were also tested against zero. A one-sample *t*-test revealed that the mean effect size was significantly greater than zero [*t*(24) = 14.95, *p* < 0.0001]. With an average effect size of ES = 0.39 (SD = 0.13, upper 5% confidence interval 0.45; lower CI 0.34), observed movement synchrony between all participants of the quartets was highly significant.

Quartet synchrony was additionally analyzed using dyadic SUSY. Within a quartet, there are six unique dyadic combinations; accordingly, further analysis explored the movement synchrony of these dyads nested in quartets. Among all the dyadic combinations (*n* = 150), *ES_noAbs_* was significantly different from zero (mean *ES_noAbs_* = 14.32, SD = 16.75; *t*(149) = 10.47, *p* < 0.0001). This supported the mv-SUSY result and showed that the synchronies between nested dyads within quartets were predominantly in-phase.

As a research question related to Hypothesis 1, we explored whether the movement synchronies of dyads that had been paired for the initial interaction would differ from their synchronies in the subsequent quartet interaction. Thus, *ES_noAbs_* of the dyads was compared with that of the same dyad in the quartet context using a paired *t*-test (*n* = 49). The difference in empirical movement synchrony was insignificant (*t*(48) = 0.08, *p* = 0.90), in other words, no evidence for a change in dyadic movement synchrony from the dyadic to the quartet setting was found.

*Hypothesis 2*: Relationship between familiarity and movement synchrony in dyads (SUSY) and quartets (mv-SUSY).

To analyze the association between movement synchrony and self-rated familiarity of interactants with one another, separate bivariate linear regressions were performed to model the dyadic and quartet interactions. For dyads (*n* = 49), *ES_noAbs_* was specified as the dependent variable and the average of both dyadic participants’ familiarity ratings as the predictor. This regression model detected a significant association between average familiarity and movement synchrony, in that the higher the degree of mutual acquaintance within a dyad, the stronger the movement synchrony (*F*(1,48) = 7.76, *p* < 0.01). This significant relationship remained stable at higher *maxlags* of 4 and 5 s (both *p* < 0.01), but in a model examining more fine-grained lags (*maxlag* = 2) only marginal effects were detected (*p* = 0.06).

A further regression model estimated whether this finding extended to quartets. Group synchrony (*ES*) between all four participants in quartets (*n* = 25) was predicted by the average group familiarity between all four participants. The model did not yield significant results (*F*(1,24) = 0.32, *p* = 0.58), not supporting an effect of mean familiarity on movement synchrony of the entire group.

Examining synchrony in quartet interactions by way of the aggregated dyadic synchronies, a further model was specified to predict changes in *ES_noAbs_* using the average familiarity of nested dyads (n = 150). The question was whether the familiarity between two individuals affected their dyadic movement synchrony when interacting in the quartet context. Given the hierarchical structure of the data, a mixed effects linear regression was used with quartet as the random effect (random intercept model). No significant effect of dyadic familiarity on movement synchrony emerged (*t*(146) = 1.52, *p* = 0.13). In an additional random slope model, again no significant familiarity effects were detected.

To further examine this relationship, individual (rather than averaged) familiarity ratings were used to capture potential asymmetries within dyads because one participant might feel more familiar with the other than vice versa. Quartet was modeled as the random effect. Movement synchrony in quartets was again not significantly predicted by individual familiarity.

To account for the nesting of individual synchrony contributions within quartets, a linear mixed-effects model was fitted with quartet as a random effect and familiarity and age as fixed effects. None of the predictors were significant, and a highly significant intra-class coefficient (ICC = 0.61) indicated that most variance was explained by quartet-level variance rather than individual-level effects.

Disregarding the variance of the quartet level, a non-hierarchical multivariate regression was conducted to explore how individual-level factors within quartets may relate to individual-level synchrony contributions (*contribES*). Familiarity was reformulated as the average of how well each participant knew all three other group members, and individual age was included as a second predictor. The best-fitting model according to Akaike’s information criterion included age, familiarity, and their interaction (age × familiarity) (*F*(3,96) = 5.25, *p* < 0.01). This model suggested positive associations of familiarity, age, and their interaction with synchrony contribution. In short, participants (n = 100) synchronized more strongly when they were older and more familiar with their respective group members.

*Hypothesis 3*: Self-reported affectivity and movement synchrony in dyads (SUSY), dyads within quartets (SUSY) and between quartets (mv-SUSY).

Prior to modelling, ratings of negative affect were found to be non-normally distributed (Shapiro–Wilk test: *W* = 0.7, *p* < 0.001) and characterized by a strong rightward skew (i.e., bottom effect; *M* = 1.2, skew = 3.1). This indicates that participants only seldomly reported any negative affect. Consequently, negative affect was not considered as an independent variable in the analysis. Positive affect ratings, on the other hand, were normally distributed (Shapiro–Wilk test: *W* = 0.90, *p* = 0.43), thus meeting prerequisites for regression analysis. The analysis below considered only positive affect derived from the three PANAS measurements.

To examine how pre- and post-interaction positive affect (PA) related to interpersonal synchrony of dyads, regression analyses were conducted using all four individual ratings of PA from PANAS 1 and PANAS 2, hence prior to and after the interaction (cf. Figure 1) as predictors of dyadic movement synchrony (*n* = 49). In this full model, affect showed no significant associations with synchrony. Subsequent modelling removed post-session predictors and introduced average dyadic familiarity as a covariate and interaction term. According to Akaike’s information criterion, the model with predictors dyadic familiarity, dyadic PA, and dyadic familiarity × dyadic PA delivered the best fit (*F*(3,45) = 5.50, *p* < 0.01). The model suggested that higher familiarity significantly predicted greater movement synchrony (*t*(45) = 3.36, *p* < 0.01) and its interaction with prior PA further enhanced this effect (*t*(45) = 2.20, *p* < 0.05). In other words, synchrony was more pronounced when partners were well-acquainted and in a positive mood before the interaction.

Regarding the quartet interactions, we assessed whether affective states influenced dyadic movement synchrony nested in quartets (*n* = 150). Two mixed-effects models were specified with PANAS 2 and PANAS 3 ratings as predictors, respectively. The first model used each participant’s PA ratings collected before the quartet interaction (PANAS 2) as predictors, with quartet as a random effect. No significant associations emerged, suggesting no influence of pre-quartet positive affect on synchrony. The second model tested the association with post-interaction affect ratings (PANAS 3), averaged by subject and dyadic permutation. Again, no significant effects were found, indicating that subjective affective states after the interaction were not associated with movement synchrony. In both models, Wald tests confirmed that roughly a third of the variance was attributable to group-level variance (ICC = 29.7%, *p* = 0.02 for the PA-pre model; ICC = 29.6%, *p* = 0.02 for the PA-post model).

To explore whether affect influenced collective group synchrony of all four participants (*ES*), two separate regression models were run using PA scores averaged per quartet. Average PA ratings of the four respective participants were tested both before (PANAS 2) and after (PANAS 3) the quartet interaction. None of the models revealed significant associations with group-level synchrony. These findings suggest that affective states, whether pre- or post-interaction, did not predict movement synchrony in multi-person settings.

## Discussion

To assess the robustness of interpersonal movement synchrony across two different participant configurations, dyads and quartets, and to evaluate the role of familiarity and affect, three hypotheses (H1–H3) were formulated. H1 predicted that body movement synchrony would occur above chance in dyadic and quartet conversations. H2 focused on the influence of familiarity, and H3 examined whether participants’ positive affect was related to the degree of synchrony. The following summary describes to which extent these hypotheses were supported.

Participants’ synchrony of body movements was found to be significant in both dyadic and quartet configurations, fully supporting H1. This was true in dyadic as well as in quartet interactions, including dyads nested within quartets, suggesting that movement synchrony generally occurred when people engaged face-to-face in pairs or in groups of four. Additionally, the extent of movement synchrony was not significantly different when dyads interacted on their own or as part of a quartet. Both are novel findings as synchrony of the same participants was not before studied in a dyadic and in a group context.

H2 was partially confirmed. Participants’ self-rated familiarity with co-participants influenced movement synchrony positively in dyads, but no effect on group synchrony (mv-SUSY) in quartets was present. In exploratory variants of the quartet analyses, individual familiarity, i.e., the average of a participant’s familiarity ratings of his or her three co-participants, was found positively related to the synchrony contribution of that person, yet only when regression models disregarded the hierarchical nature of the dataset, thus the variance of the quartet level. In other words, the better participants knew each other, the stronger were they synchronized in the dyadic configuration, whereas this effect did not generalize to quartet-level or nested dyadic synchrony within quartets.

Regarding H3, of the three affect ratings sampled in the course of the study, the initial ratings of positive affect, prior to the dyadic interactions, were weakly associated with increased movement synchrony during dyadic conversations, yet only as part of an interaction effect with familiarity. In quartets, no associations between positive affect and movement synchrony were found. In sum, the analyses do not support H3. Positive affective states measured before or after interactions did not exhibit significant main effects on movement synchrony in either dyadic or quartet interactions.

Altogether, these results constitute clear evidence that informal conversations on everyday topics, such as preferred or disliked dishes, are characterized by movement synchrony. In this study, this was true for two participant configurations, i.e., conversation in dyads, and ensuing conversation in four-person groups. Two different algorithms to quantify synchrony, dyadic surrogate synchrony (SUSY) based on the cross-correlation function, and multivariate synchrony (mv-SUSY) based on the eigenvalues of the correlation matrix, were implemented and both consistently pointed toward significant synchrony. Additionally, the degree of dyadic synchrony remained stable when these dyads later became part of a group. Hence, a change of the social configuration did not change movement synchrony between participants—neither did the previously, already attuned dyads show stronger synchrony in the new environment, nor was their synchrony attenuated owing to the new social demands of the group context. Thus, although one might expect the movement synchrony of nested dyads to weaken in the presence of additional co-participants in a more complex participation framework, their interpersonal synchrony remained robust.

Two predictors were analyzed with regard to their associations with synchrony, the familiarity of participants and participants’ affective states prior to and after the conversation interactions. Familiarity was supportive of synchrony especially in the initial dyadic interactions, yet not in the ensuing group context (nested dyads and quartets). This decreasing impact may be caused by the fixed sequence of dyadic conversations followed by quartet conversations (see Figure 1), where familiarity had an impact mainly on the initial interaction. This link between synchrony and familiarity was expected as numerous findings have shown that synchrony is related to social bonding (Ramseyer and Tschacher, 2011; Hale et al., 2019), affiliation (Hove and Risen, 2009) and personal similarities (termed ‘homophily’: Laakasuo et al., 2020; Chierchia and Coricelli, 2015). The absence of an effect of familiarity on movement synchrony in nested dyads or on the collective synchrony of quartets may reflect the additional demands of the quartet setting. Shifting participation structures, parallel conversational threads, and the need to monitor multiple co-participants may have attenuated dyad- and group-level effects. Moreover, since even initially unfamiliar nested dyads had participated in a face-to-face conversation prior to the quartet interaction, they may already have become sufficiently familiar with one another, thereby further reducing the impact of familiarity in the quartet condition.

The present study did not confirm the association between synchrony and affect previously found in conversations between strangers (Tschacher et al., 2014). This may have been caused by the conditions when participants filled out the PANAS questionnaires. Ratings were not done separately but in the dyad or group in which the upcoming conversation would take place. Thus, it is possible that affect ratings were biased by familiarity. This would be in line with our finding that positive affect prior to the interaction amplified synchrony only in familiar dyads, indicating that affect was not a stand-alone predictor but acted only as an interaction effect with relational factors. Positive affect fosters prosocial orientation toward interaction partners (Aknin et al., 2018). Our results suggest that positive affect may strengthen movement synchrony particularly when relational ties are already present. However, because affect ratings prior to the quartet conversation were not associated with synchrony, any extension of this mechanism to multi-party settings remains speculative. In quartets, the multiple degrees-of-freedom and the tendency to report socially desirable positive affect in group contexts (see below) may have limited an observable relationship of affective states and movement synchrony.

Regarding methodological considerations identified in the analyses, the multi-level structure of the data may have influenced the results. For example, computing collective synchrony across all four participants in each quartet and averaging familiarity scores at the group level constitute relatively coarse aggregation steps that may have obscured intra-group and individual variability, thereby reducing the model’s sensitivity to specific effects. Notably, a more fine-grained analysis at the individual level revealed that familiarity influenced participants’ contributions to synchrony within quartets. Specifically, the degree to which a participant knew the other group members affected how strongly their movements contributed to overall synchrony. This association was not observed for overall group synchrony, possibly reflecting the aggregation of data in that analysis.

Another aspect to warrant consideration is the adapted use of the PANAS surveys. PANAS surveys were administered before and after each interaction phase, with participants completing them in co-presence at a shared table. This procedure was selected to minimize unnecessary participant movement^7^, accommodate available laboratory conditions, and align with the broader mixed-methods framework of the project (integration of Conversation Analysis and Interactional Linguistics for qualitative studies on spoken language, gaze and synchrony). The co-presence of other participants may have influenced affectivity ratings, owing to social desirability, such as indicating positive affect while minimizing reports of negative affect^8^, or, on rare occasions, through informal discussion of specific PANAS items. In addition, PANAS items were not presented in random order, with all positive adjectives preceding the negative ones, which may have shaped responses in later survey administrations. The correlations detected by statistical models between movement synchrony and the affectivity ratings were observed only for the first PANAS administration, suggesting that these initial ratings provided a reliable measure for interpreting the reported effects. Therefore, the finding that positive affect prior to the initial dyadic interaction predicted movement synchrony may be interpreted as reliable.

A further aspect that merits consideration concerns the approach to controlling familiarity within participant configurations. Although recruiting small groups of students from the same faculty made participant familiarity likely, the semi-experimental design did not include systematic counterbalancing of familiarity across dyads. As a result, differences in synchrony between dyads could partly reflect varying familiarity levels, providing for a higher degree of ecological validity while decreasing experimental control conditions. However, since familiarity was an interval-scaled predictor of movement synchrony, the design did not require balanced categorical groupings (e.g., strangers vs. friends). Moreover, familiarity ratings were relatively evenly distributed across the scale, reducing potential bias.

The last methodological aspect concerns the degree of flexibility allowed in the task execution. Participants were asked to perform a “fun task” (Tschacher et al., 2014, p. 3) of planning a hypothetical five-course dinner together. This task was chosen because it has been shown to be associated with high movement synchrony among strangers (ibid.). In the present study, participants were told that deviations from the topic were acceptable. Consequently, some groups may have approached the task as casual conversation rather than a joint problem-solving activity, which may have affected the extent of mutual engagement and movement synchrony.^9^ While such flexibility could be viewed as a methodological limitation from an experimental psychology perspective, it was a deliberate choice to maintain ecological validity and ensure that the recorded interactions remained suitable for studies in Conversation Analysis and Interactional Linguistics. Restricting participants’ talk to narrow, externally defined, goal-oriented discussions might have reduced spontaneity and altered natural, social interactional dynamics. As Gordon et al. (2025) argue, interpersonal synchrony is flexible, with multiple factors shaping how individuals align with or diverge from others. While our quantitative analyses capture movement synchrony across participant configurations, they reveal little about its interactional function. These methods may be limited in assessing, for instance, whether highly synchronized participants experienced greater bonding, as suggested by evolutionary accounts (see above), or whether they tended to diverge.

To date, it remains a desideratum to examine spontaneous (non-induced) interpersonal synchrony by integrating qualitative, sequential analysis of verbal and embodied interaction with quantitative measures of synchrony. Research on temporality in social interaction (Deppermann and Streeck, 2018; Meyer et al., 2017; Streeck et al., 2011; Stukenbrock, 2023) has shown that interpersonal synchrony is not a clearly bounded, purely simultaneous event, but rather an interactional accomplishment that is shaped by micro-projections within, between, and across utterances and actions (Auer, 2005, 2024; Goodwin, 1980; Streeck, 1993; Stukenbrock, 2018b). This holds also for the movement synchrony in our sample: the synchrony had already been ‘in the making’ before it became measurable. However, the synchrony-computation algorithms used in this study yield an aggregated synchrony measure for a defined time period and thus do not capture its moment-by-moment build-up.

Further challenges concern the integrated analysis of multiple modalities (for discussion, see Gordon et al., 2025), as well as the further integration of spoken language—the species-specific means through which humans co-orient, interact, and collaborate in social encounters. Although language is occasionally included in the research design (cf. Wiltshire et al., 2020, for review; Shockley et al., 2003), these approaches do not account for the sequential organization and temporal unfolding of spoken language in social interaction. Future work should link quantitative findings on the temporal dynamics of nonverbal synchrony to detailed analyses of how participants’ verbal and embodied actions are temporally attuned in socially accountable ways.

Taken together, our methodological considerations highlight the challenges involved in studying naturalistic social interaction in multi-party settings while ensuring analytical rigor. The present study aimed to balance ecological validity with experimental control, seeking to achieve both statistical robustness and qualitative interpretability for follow-up studies. Despite the outlined constraints, this study provides robust evidence for a link between movement synchrony, familiarity and affect, thus contributing to a more comprehensive understanding of humans’ mutual attunement in naturalistic interactions.

## Conclusion

This study examined interpersonal synchrony of body movement in spontaneous conversations, comparing dyads and quartets. Relating synchrony to participants’ familiarity and positive affect ratings, we found that movement synchrony was present in both configurations—at the collective (quartet) level and between dyadic participants. Moreover, we observed that dyadic synchrony remained robust when these dyads later interacted as part of the quartet. Importantly, higher familiarity was positively associated with higher synchrony in dyads, contributing to the still limited evidence linking familiarity and synchronization phenomena (Latif et al., 2014; Rennung and Göritz, 2016; Gordon et al., 2025). By contrast, no corresponding main effects of familiarity emerged for quartet-level synchrony. Although affect analyses were limited, they revealed that more familiar dyads displayed higher synchrony when they reported higher pre-interaction positive affect (i.e., affect affected synchrony), suggesting that affect did not operate as a stand-alone predictor but in interaction with relational factors. This pattern complements Tschacher et al. (2014), who observed that stranger dyads reported more positive post-interaction affect when they had been more synchronized (i.e., synchrony affected affect). Taken together, these findings suggest that interpersonal synchrony in informal conversation is not restricted to dyadic settings and, importantly, remains robust when the same dyads are embedded in more complex participation frameworks (quartets). They also show that familiarity—and to some extent positive affect—shapes movement synchrony primarily in dyadic interaction, underscoring the value of examining both dyadic and quartet configurations with the same participants. Future research should further disentangle the temporal and motivational dynamics that link synchrony with social experience across diverse interactional contexts.

## Data Availability

The raw data supporting the conclusions of this article will be made available by the authors upon request.

## Footnotes

1 Tschacher et al. (2023: 10) note: “In the psychological field of nonverbal synchrony, terminology is still inconsistent: attunement, concordance, coupling, entrainment, mimicry, coordination, adaptation, synchronicity, and more. ‘Synchrony’ (Greek: ‘together in time’) is the increasingly prevailing term to denote the phenomenon as this term makes no causal assumptions, does not claim intentionality, is semantically neutral, and not by convention used in other contexts.”

2 A sensor-free methodology for capturing body movement from video recordings is Motion Energy Analysis (MEA; Ramseyer, 2020), which can be complemented by data from wearable sensors to record eye movements or physiology. A well-established set of methods for the computation of synchrony based on such data are surrogate synchrony (SUSY; Tschacher and Meier, 2020) and multivariate surrogate synchrony (mv-SUSY; Meier and Tschacher, 2021). Other, non-surrogate based methods for the quantification of synchrony include, for instance, Kuramoto models of phase-synchronization (Frank and Richardson, 2010) or multidimensional cross-recurrence quantification analysis (Wallot, 2019).

3 Induction synchrony is “experimentally induced or instructed behavioral synchrony [and] given especially in the setting of classical concerts, where there is little interaction between members of the audience” (p. 2) but rather engagement with the musical performance.

4 Verbatim instructions were: “We would like you to have a conversation. To make this easier for you, we are giving you the following task: Discuss a 5-course meal where you do not like a single dish – essentially, an anti-menu. Feel completely free; this will not be evaluated. You are also welcome to stray from the topic – the more spontaneous, the better.”

5 Verbatim instructions were: “Now we are turning the tables. Talk about a 5-course meal made up of dishes you all like.”

6 The number of generated surrogates is calculated as *n(n − 1)*, that is, by multiplying the total number of segments by the total number minus one (Tschacher and Meier, 2020). This yields the unique cross-pairings of mismatched segments from the original dyadic time series. Accordingly, the number of surrogates depends on the duration of the interaction (i.e., the number of segments). Across the 49 dyads analyzed here, the average number of surrogates was 718.

7 As participants wore mobile eye-tracking glasses for follow-up studies, movement may cause minor head unit displacement, potentially introducing measurement noise or partial data loss.

8 This is consistent with the bottom-effect observed in the negative affect ratings.

9 Tschacher et al. (2014: 9) observed that competitive conversation-tasks were associated with higher movement synchrony between stranger dyads than cooperative conversation tasks (see also Gordon et al., 2025: 690).
